# ^1^H NMR-Based Metabolomics Coupled With Molecular Docking Reveal the Anti-Diabetic Effects and Potential Active Components of *Berberis vernae* on Type 2 Diabetic Rats

**DOI:** 10.3389/fphar.2020.00932

**Published:** 2020-06-19

**Authors:** Qi Li, Chengcheng Zhao, Yunsen Zhang, Huan Du, Tong Xu, Xinmei Xu, Jing Zhang, Tingting Kuang, Xianrong Lai, Gang Fan, Yi Zhang

**Affiliations:** ^1^School of Pharmacy, Chengdu University of Traditional Chinese Medicine, Chengdu, China; ^2^School of Ethnic Medicine, Chengdu University of Traditional Chinese Medicine, Chengdu, China

**Keywords:** *Berberis vernae*, herbal medicine, type 2 diabetes, metabolomics, molecular docking

## Abstract

The dried stem bark of *Berberis vernae* C.K.Schneid., known as “Xiao-bo-pi” in Chinese, is a representative anti-diabetic herb in traditional Tibetan medical system. However, its anti-diabetic mechanisms and active components remain unclear. In this study, ^1^H NMR-based metabolomics, biochemistry assay, molecular docking, and network analysis were integrated to evaluate the anti-diabetic effects of *B. vernae* extract on type 2 diabetic rats, and to explore its active components and underlying mechanisms. Diabetes was induced by high-fat diet and streptozotocin. After 30 days of treatment, *B. vernae* extract significantly decreased the serum levels of fasting blood glucose, insulin, insulin resistance index, glycated serum protein, TNF-α, IL-1β, and IL-6, whereas significantly increased the serum levels of insulin sensitivity index in type 2 diabetic rats. A total of 28 endogenous metabolites were identified by ^1^H NMR-based metabolomics, of which 9 metabolites that were changed by diabetes were significantly reversed by *B. vernae* extract. The constructed compound-protein-metabolite-disease (CPMD) interaction network revealed the correlation between chemical constituents, target proteins, differential metabolites, and type 2 diabetes. Ferulic acid 4-*O*-*β*-D-glucopyranoside, bufotenidine, jatrorrhizine, and berberine showed good hit rates for both the 30 disease-related proteins and 14 differential metabolites-related proteins, indicating that these four compounds might be the active ingredients of *B. vernae* against type 2 diabetes. Moreover, pathway analysis revealed that the anti-diabetic mechanisms of *B. vernae* might be related to its regulation of several metabolic pathways (e.g., butanoate metabolism) and disease-related signal pathways (e.g., adipocytokine signaling pathway). In summary, *B. vernae* exerts a significant anti-diabetic effect and has potential as a drug candidate for the treatment of type 2 diabetes.

## Introduction

Diabetes mellitus is a group of metabolic diseases characterized by hyperglycemia resulting from defects in insulin secretion, insulin action, or both (American Diabetes Association, 1997). According to the 8th edition of International Diabetes Federation (IDF) Diabetes Atlas released by IDF in 2017, there are currently 425 million adults (20–79 years old) with diabetes, and the number of patients is still increasing (International Diabetes Federation, 2017). It is estimated that there will be 629 million diabetic patients by 2045. Therefore, it is of great significance to find effective drugs to treat diabetes. Searching for good anti-diabetic drugs from traditional natural medicines has attracted increasingly attention.

Traditional Tibetan medicine (TTM), one of the world’s oldest known medical systems, has a long history of more than 2,000 years (Li et al., 2018). In the long-term clinical practice, TTM has accumulated rich experience in the treatment of diabetes mellitus. The dried stem bark of *Berberis vernae* Schneid., known as “Xiao-bo-pi” in Chinese, is a commonly used herb for the treatment of type 2 diabetes (Chinese Pharmacopoeia Commission, 1995; Zhang et al., 2013). In TTM monographs and drug standards, such as “*Drug Standards of Tibetan Medicines*,” “*Lan Liu Li*,” and “*The Four Medical Tantras*” (Yutuo, 1987; Chinese Pharmacopoeia Commission, 1995; Disi, 2012), Xiao-bo-pi is described as bitter in flavor and cold in property, and can treat dysentery, frequent urination, nephritis, and diabetes. Modern pharmacological study has demonstrated that Xiao-bo-pi has an obvious hypoglycemic effect on alloxan-induced diabetic mice (Zhang et al., 2013). However, its anti-diabetic mechanisms are not well elucidated. Moreover, the main chemical constituents of *Berberis* plants are recognized as some alkaloids, such as berberine, magnoflorine, and jateorrhizine (Belwal et al., 2020; Bhardwaj and Kaushik, 2012). They have been proven to possess anti-inflammatory, anti-diabetic, and antioxidant activities (Grycova et al., 2007; Tang et al., 2006; Neag et al., 2018). However, whether there are other active ingredients with anti-diabetic effect in *B. vernae* remains to be further studied.

Metabolomics has attracted a great deal of interest in recent years because of its holistic characteristics. In the past decade, ^1^H NMR-based metabolomics approach has been widely used to evaluate the pharmacological activity and molecular mechanisms of traditional herbal medicines (Abas et al., 2016; AL-Zuaidy et al., 2017). Molecular docking, a computer virtual docking method, has been successfully used in the research of active ingredients and potential targets of traditional Chinese medicine (Gu et al., 2011; Chen et al., 2014). In this study, we applied conventional biochemistry assay, ^1^H NMR-based metabolomics, high-performance liquid chromatography (HPLC), molecular docking, and network analysis methods to evaluate the anti-diabetic effects of *B. vernae* on type 2 diabetic rats induced by high-fat diet and streptozotocin (STZ), and more importantly, to reveal its underlying pharmacological mechanisms and active compounds. These studies will be beneficial for the development and clinical application of *B. vernae* in the treatment of diabetes.

## Materials and Methods

### Chemicals and Medicinal Materials

Jatrorrhizine, palmatine, and berberine were obtained from Scientist Biotechnology Co., Ltd. (Chengdu, China). Magnoflorine was provided by Chengdu Must Biotechnology Co., Ltd. (Chengdu, China). Bufotenidine and ferulic acid 4-*O*-*β*-D-glucopyranoside were isolated and purified from Xiao-bo-pi by our research team (Li Q. et al., 2019), and their structures were unambiguously identified by ^1^H NMR and ^13^C NMR methods. The purity of each standard compound was over 98%. STZ was purchased from Sigma-Aldrich Co., St. (Louis, MO, USA). Metformin hydrochloride tablets and D_2_O were purchased from Sino-American Shanghai Squibb Pharmaceuticals Ltd. (Shanghai, China) and Cambridge Isotope Laboratories, Inc. (USA), respectively. Both NaH_2_PO_4_ and K_2_HPO_4_ were provided by Sinopharm Chemical Reagent Co., Ltd. (Shanghai, China). All other chemicals used were of analytical grade.

The dried stem bark of *B. vernae* was collected and identified by DNA barcoding method as established in our previous study (Feng et al., 2018). The voucher specimen (No. XBP0810) was deposited in the School of Ethnic Medicine, Chengdu University of Traditional Chinese Medicine, Chengdu, China.

### Preparation of *B. vernae* Extract

The dried stem bark of *B. vernae* was cut into small pieces, and extracted three times with pure water (1:10, w/v) by decocting (each extraction period lasted 1 h). Subsequently, the filtrate was mixed and evaporated to dryness. The extract was dissolved in physiological saline before use.

### HPLC Analysis of *B. vernae* Extract

Chromatographic separation was performed on an Agilent 1260 Series HPLC system equipped with a quaternary pump, temperature-controlled autosampler, and diode array detector recorded from 200 to 400 nm (Agilent technologies, Germany). A WondaSil C_18_ column (250 mm × 4.6 mm, 5 μm, Shimadzu, Japan) was used. The UV spectra were recorded between 200 and 400 nm, and a wavelength channel of 270 nm was applied for quantitative purposes. The mobile phase was 0.2% phosphoric acid in water (A) and acetonitrile (B) with a gradient elution program of 5–7% B at 0–10 min, 7–12% B at 10–12 min, 12–12% B at 12–28 min, and 12–25% B at 28–31 min. The flow rate was 1.0 ml/min, and the injection volume was 5 μl.

*B. vernae* extract (0.1 g) was accurately weighed into a clean Erlenmeyer flask and extracted with 20 ml of hydrochloric acid: 70% methanol (1:100) by ultrasonication for 30 min. The sample solution was filtered through a 0.22 μm nylon filter membrane. Quantitative analysis of six constituents in *B. vernae* extract was carried out according to our previously reported method (Chen et al., 2014). The concentrations of bufotenidine, ferulic acid 4-*O*-*β*-D-glucopyranoside, magnoflorine, jatrorrhizine, palmatine, and berberine were calculated using the established calibration curves made from the standard compounds. Quantitative determination of the six compounds was done in duplicate.

### Animals

Male Sprague-Dawley (SD) rats (160–180 g) were procured from Chengdu Dossy Experimental Animals Co., Ltd. (Chengdu, China). The animals were housed in individual cages and maintained under controlled room temperature (25 ± 2°C) and humidity (55 ± 5%) with 12:12 h light and dark cycle. All the rats were provided with commercially available rat normal pellet diet (NPD) (Chengdu Dossy Experimental Animals Co., Ltd., Chengdu, China) and water *ad libitum*, prior to the dietary manipulation. The studies were approved by the animal ethics committee of Chengdu University of Traditional Chinese Medicine, and were in conformity with the National Institute of Health (NIH) guidelines for the care and use of laboratory animals.

### Development of High-Fat Diet and STZ Induced Type 2 Diabetic Rats

The rats were allocated into two dietary regimens by feeding either NPD or high-fat diet (HFD) *ad libitum*. The HFD consisting of 70% NPD, 16% sucrose, 12% lard, 1% cholesterol, and 1% sodium cholate (Shao and Cai, 2014) were provided by Chengdu Dossy Experimental Animals Co., Ltd. (Chengdu, China). After the 4 weeks of dietary manipulation, the group of rats fed with HFD was injected intraperitoneally (i.p.) with a low dose of STZ (45 mg/kg) (Gao, 2018), while the respective control rats fed with NPD were intraperitoneally given the equivalent volume of vehicle citrate buffer (0.1 M, pH 4.4). After 72 h, rats with fasting blood glucose (FBG) ≥ 11.0 mmol/L were taken for the experiment. The rats were allowed to continue to feed on their respective diets until the end of the study.

### Experimental Design

The experimental animals were divided into four groups, each group comprising eight rats, as detailed follows: Group 1 (normal) served as normal control rats; Group 2 (model) served as diabetic model control rats; Group 3 (metformin) and Group 4 (SY) served as diabetic rats administered with metformin hydrochloride tablets (0.25 g/kg b.w.) (Kang et al., 2018) and *B. vernae* extract (0.84 g/kg b.w.) (Zhang et al., 2013) in aqueous suspension orally for 30 days, respectively. The dosage was adjusted every week according to any change in body weight to maintain similar dose per kg body weight of rat over the entire period of study for each group. At the end of the experimental period, the rats were fasted overnight. The blood was collected in tubes and serum was separated by centrifugation at 3,500 rpm for 10 min, stored at −80°C before biochemical assay. The serum was further centrifuged at 11,000 rpm for 10 min, and the supernatant was collected and stored at −80°C before ^1^H NMR analysis.

### Biochemical Analysis

After 30 days of treatment, rats were fasted for 12 h. FBG was measured with Sannuo glucometer (Sinocare Inc., China). The levels of glycated serum protein (GSP), insulin (INS), tumor necrosis factor–α (TNF–α), and interleukin–6 (IL–6) were evaluated by commercial rat ELISA kit (Shanghai Enzyme-linked Biotechnology Co., Ltd., China). The levels of interleukin–1β (IL–1β) were evaluated by commercial rat ELISA kit (MultiSciences Biotech Co., Ltd, China). Homeostasis model assessment of insulin resistance (HOMA-IR) and insulin sensitivity index (ISI) were calculated using the following formulas (Chen et al., 2018; Li J. P. et al., 2019) [20, 21]: HOMA-IR = INS (mIU/L) × FBG (mmol/L)/22.5; ISI = Ln [1/(FBG × INS)].

### ^1^H NMR Analysis

Before ^1^H NMR analysis, the supernatant of serum samples were thawed at room temperature. 200 μl supernatant was mixed with 200 μl phosphate buffer solution (pH 7.4, 45 mM) and 200 μl D_2_O into tube. The mixture was centrifuged at 12,000 rpm at 4°C for 10 min, and 550 μl supernatant of the mixture was transferred to a 5 mm NMR tube. The ^1^H NMR measurements of samples were performed using a 600 MHz Varian NMR spectrometer (Varian Inc., Palo Alto, CA, USA), functioning at frequency of 599.93 MHz and maintained at 25°C. A standard water-suppressed one dimensional NMR was obtained using the Carr-Purcell-Meiboom-Gill pulse sequence (64 scans). The acquisition time of each ^1^H NMR spectrum was 1.5 s.

### Data Processing and Statistical Analysis

The ^1^H NMR spectra was manually subjected to Fourier transform, phase and baseline correction using MestReNova software (Version 9.0.1, Mestrelab Research, Santiago de Compostella, Spain). Lactate with a chemical shift at *δ* 4.11 was used as a reference of the serum spectrum. The spectral regions of *δ* 0.5–9.0 were integrated in 0.002 ppm intervals. The regions contained residual water signals (*δ* 4.68–5.00) were removed prior to data normalization. The normalized integral data were subjected to multivariate pattern recognition analysis using the SIMCA-P software (Version 14.1, Umetrics, Umea, Sweden). The ^1^H NMR data were analyzed by principal component analysis (PCA) and partial least squares discriminant analysis (PLS-DA) to differentiate each group. Orthogonal projections to latent structures discriminant analysis (OPLS-DA) was used to find differential metabolites from the S-plots. All PLS-DA and OPLS-DA models were cross-validated using 200 permutation tests by default. The quality of the models was assessed by the parameters R^2^, and the predictability was described by Q^2^. Clustering heatmap was generated using MetaboAnalyst 4.0 (https://www.metaboanalyst.ca). The color of each section change from dark blue through crimson in the heatmap corresponds to a change from low to high for each metabolite concentration.

All data were presented as mean ± SEM. One-way ANOVA followed by Dunnett’s test was performed using GraphPad Prism (Version 5.0, GraphPad Software Inc., San Diego, CA, USA). A value of *P* < 0.05 was considered statistically significant.

### Molecular Docking and Network Construction

Metscape, a plugin for Cytoscape (Version 3.7.0), was used to search for candidate proteins associated with differential metabolites (Gao et al., 2010). Candidate targets related to type 2 diabetes were searched in the DrugBank (https://www.drugbank.ca/) and Therapeutic target database (TTD) (http://db.idrblab.net/ttd/). The corresponding PDB IDs of these targeted proteins were obtained from UniProt (https://www.uniprot.org/). The protein structures without PDB ID were obtained by homology modeling using Maestro software (Version 11.1, Schrodinger, LLC, New York, 2017), and the established structure was evaluated by the Ramachandran plot, and proteins with more than 90% of the residues located in the optimal area and the maximum allowable area were taken for the next experiment. All target proteins are expressed by PDB ID or gene name.

All compounds were first screened by the Lipinski’s rule of five, which is molecular weight lower than 500 Da, number of donor hydrogen bonds less than 5, number of acceptor hydrogen bonds less than 10, and fat water partition coefficient miLog *P* lower than 5 (Nogara et al., 2015; Ma et al., 2016). Compounds meeting the above rules are further used for molecular docking. Their structures were obtained from PubChem (https://pubchem.ncbi.nlm.nih.gov/) or drawn in the ChemDraw (Version 7.0). Molecular docking was performed using Maestro software. The greater the absolute value of the docking scores between the compound and the target protein, the higher the docking affinity. Compounds with an absolute value of docking scores >5.0 were considered to be potential active components.

A compound-protein-metabolite-disease (CPMD) network was constructed using Cytoscape to show the correlation between chemical constituents of *B. vernae*, target proteins, differential metabolites, and type 2 diabetes. In addition, clustering heatmap of docking scores was generated using MultiExperiment Viewer software (MeV, version 4.8.1). The color in the heatmap changes from green to red, reflecting the change from low to high of the docking score of each compound with the target protein. DAVID Bioinformatics Resources 6.8 (https://david.ncifcrf.gov/) was used to enrich the KEGG pathway of target protein.

## Results

### Effects of *B. vernae* Extract on Biochemical Parameters

The effects of *B. vernae* extract on serum FBG, INS, HOMA-IR, GSP, TNF-α, IL-1β, IL-6, and ISI levels in type 2 diabetic rats are shown in **Table 1**. As expected, all diabetic rats showed an increase in FBG and GSP. In addition, the levels of HOMA-IR, INS, TNF-α, IL-1β, and IL-6 were significantly higher (*P* < 0.01), whereas the ISI was significantly lower (*P* < 0. 01) in the serum of the diabetic rats than those in the normal rats. Additionally, our results revealed that *B. vernae* extract or metformin treatment for 30 days significantly decreased (*P* < 0.01) serum FBG, INS, HOMA-IR, GSP, TNF-α, IL-1β, and IL-6 levels, whereas increased serum ISI levels (*P* < 0.01) in type 2 diabetic rats, compared with the model group.

**Table 1:** Effects of *B. vernae* extract on several biochemical indicators.

Groups	FBG (mmol/L)	INS (mIU/L)	HOMA-IR	ISI	GSP (mmol/L)	TNF-α (pg/mL)	IL-1β (pg/mL)	IL-6 (pg/mL)
Normal	3.91 ± 0.29^**^	15.76 ± 1.87^**^	2.82 ± 0.51^**^	−4.05 ± 0.16^**^	1.17 ± 0.09^**^	125.31 ± 17.61^**^	124.26 ± 22.09^**^	120.66 ± 13.26^**^
Model	21.70 ± 0.56	27.08 ± 3.10	26.18 ± 3.24	−6.33 ± 0.12	1.76 ± 0.06	299.03 ± 25.46	363.83 ± 42.05	257.66 ± 17.78
Metformin	16.11 ± 0.68^**^	18.76 ± 1.41^*^	13.43 ± 1.15^**^	−5.69 ± 0.08^**^	1.40 ± 0.05^**^	156.04 ± 14.47^**^	179.32 ± 31.43^**^	151.94 ± 15.04^**^
SY	17.91 ± 0.44^*^	18.81 ± 1.12^*^	14.91 ± 0.84^**^	−5.81 ± 0.05^*^	1.47 ± 0.06^*^	189.42 ± 24.69^**^	196.53 ± 28.80^**^	166.06 ± 17.13^**^

* and ** indicate P ＜ 0.05 and P ＜ 0.01, respectively.

### Effects of *B. vernae* Extract on Serum Metabolites by ^1^H NMR Metabolomics Analysis

**Figure 1:**
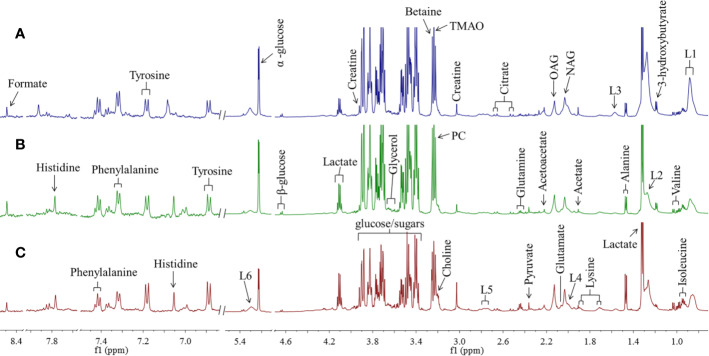
Representative ^1^H NMR spectra of rat serum from SY group **(A)**, model group **(B)**, and normal group **(C)** with metabolites labeled. TMAO, trimethylamine N-oxide; OAG, O-acetyl glycoproteins; NAG, N-acetyl glycoproteins; L1~L2, LDL/VLDL; L3~L6, lipids; PC, phosphorylcholine.

According to the chemical shifts published in the literature (Fan, 1996) databases HMDB (http://www.hmdb.ca) and BMRB (http://www.bmrb.wisc.edu), 28 metabolites were identified. Representative ^1^H NMR spectra of rat serum from normal, model, and SY groups are displayed in **Figure 1** with metabolites labeled. For detail observation of metabolite differences caused by the intervention of *B. vernae* extract, ^1^H NMR spectra were subjected to multivariate analysis.

**Figure 2:**
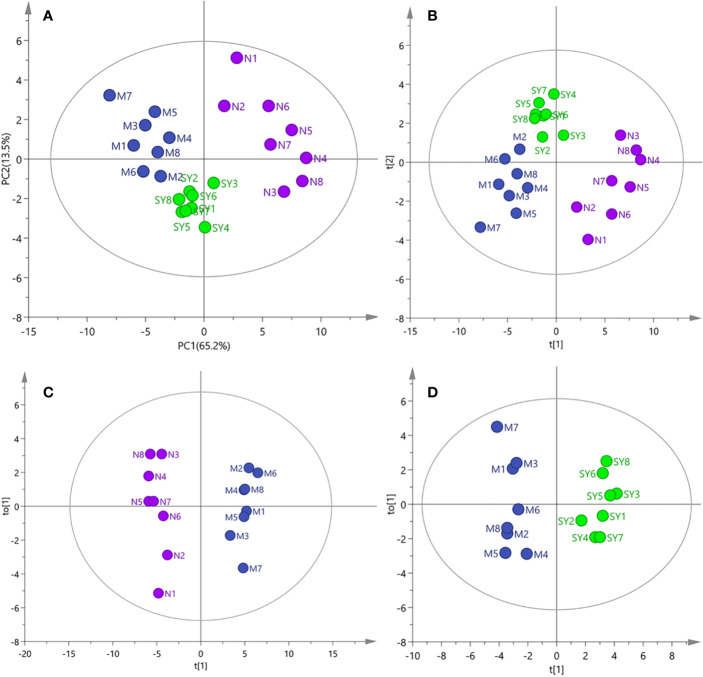
PCA, PLS-DA, and OPLS-DA analysis of ^1^H NMR data of all samples (N, normal group; M, model group; SY, *B. vernae* group). **(A)** PCA score plot of all samples; **(B)** PLS-DA score plot of all samples; **(C)** OPLS-DA score plot of normal and model group samples; **(D)** OPLS-DA score plot of model and SY group samples.

The results of PCA, PLS-DA, and OPLS-DA (**Figure 2**) showed that the serum samples of rats in normal, model, and SY groups were clearly separated, suggesting that type 2 diabetes caused significant changes in metabolites. However, the score plots of PCA and PLS-DA clearly showed that after 30 days of consecutive treatment, the SY group was closer to the normal group than the model group, which may be due to the fact that *B. vernae* extract effectively restored the metabolic disorders caused by diabetes.

**Figure 3:**
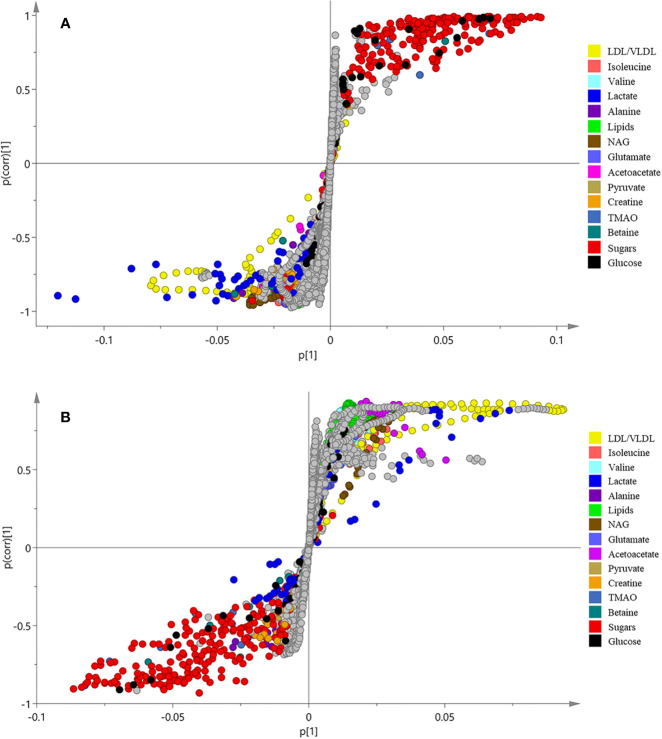
S-plots of OPLS-DA analysis of ^1^H NMR data from normal and model groups **(A)** as well as model and SY groups **(B)**.

In order to find the differential metabolites between the groups, S-plots of OPLS-DA (**Figure 3**) was further observed. The point farther from the center of S-plots is considered to contribute more to the classification, and the different color-coded points in the S-plots represent different metabolites. The differential metabolites detected and their *P*-values are summarized in **Table 2**. A total of 14 differential metabolites were assumed as potential biomarkers of diabetes, and 9 metabolites were adjusted after treatment, which are thought to be related to the anti-diabetic effects of *B. vernae*. Of which, six metabolites including low density lipoprotein/very low density lipoprotein (LDL/VLDL), isoleucine, valine, lipids, N-acetyl glycoproteins (NAG), and acetoacetate were increased, whereas three metabolites containing trimethylamine N-oxide (TMAO), betaine, and glucose were decreased after treatment with *B. vernae* extract for 30 days.

**Table 2:** Potential biomarkers in rat serum and their variations among groups.

No.	Metabolites	Chemical shift (ppm)^a^	Assignment	Model *vs.* Normal^b^	SY *vs.* Model^b^
1	LDL/VLDL	0.85 (br), 1.25 (br)	–CH_3_, –CH_2_	↓ ^***^	↑ ^***^
2	Isoleucine	0.95 (t)	–CH_3_	↓ ^***^	↑ ^***^
3	Valine	0.98 (d), 1.03 (d)	γ–CH_3_, γ–CH_3_	↓ ^***^	↑ ^**^
4	Lipids	1.56 (br)	–CH_2_–CH_2_–C=O	↓ ^***^	↑ ^*^
5	NAG	2.03 (s)	–CH_3_	↓ ^***^	↑ ^*^
6	Acetoacetate	2.22 (s)	–CH_3_	↓ ^***^	↑ ^***^
7	TMAO	3.24 (s)	–CH_3_	↑ ^***^	↓ ^***^
8	Betaine	3.25 (s)	–CH_2_	↑ ^***^	↓ ^***^
9	Glucose	4.64 (d), 5.23 (d)	1–CH	↑ ^***^	↓ ^***^
10	Lactate	1.32 (d), 4.11 (q)	β–CH_3_, α–CH	↓ ^***^	–
11	Alanine	1.47 (d)	β–CH_3_	↓ ^***^	–
12	Glutamate	2.05 (m)	β–CH_2_	↓ ^***^	–
13	Pyruvate	2.36 (s)	β–CH_3_	↓ ^***^	–
14	Creatine	3.03 (s), 3.92 (s)	–CH_3_, –CH_2_	↓ ^***^	–

^a^ s, singlet; d, doublet; q, quartet; m, multiplet; br, broad resonance.

^b^ Metabolites with “↑/↓” means increased/decreased, “–” means no significant differences. ^*^, ^**^, and ^***^ indicate P < 0.05, P < 0.01, and P < 0.001, respectively.

**Figure 4:**
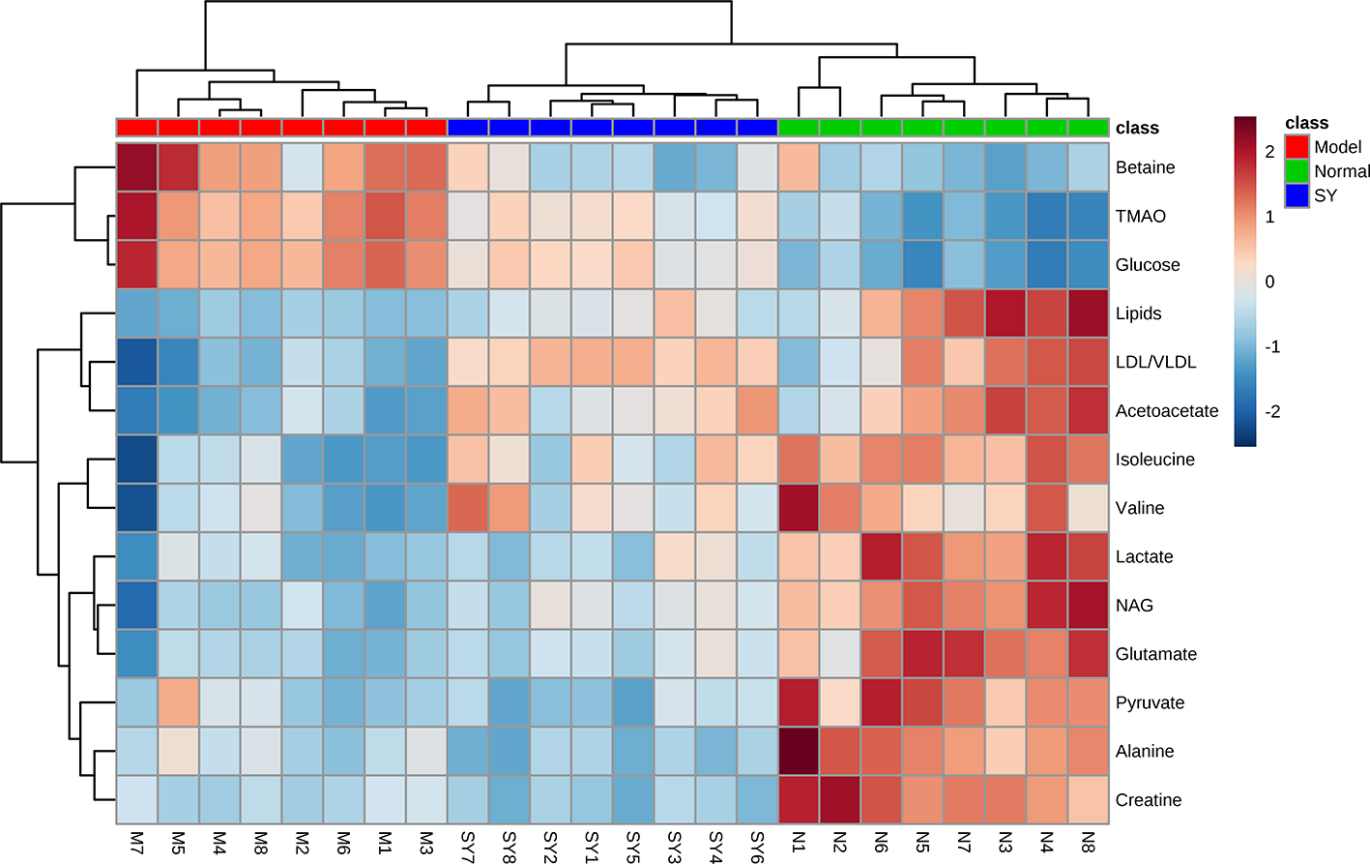
Heatmap visualization of key metabolite expression in the three groups (dark blue through crimson corresponding to a progressive increase in concentration). Each column represents a sample and each row represents a metabolite. N, normal group; M, model group; SY, *B. vernae* group.

Furthermore, clustering heatmap (**Figure 4**) was used to more visually observe the changes of 14 differential metabolites in the three groups. The results showed that the rat serum samples were significantly clustered into three groups, indicating that the serum metabolites of the three groups were obviously different, which echoed the results of PCA, PLS-DA, and OPLS-DA.

### Chemical Constituents of *B. vernae* Extract Determined by HPLC

**Figure 5:**
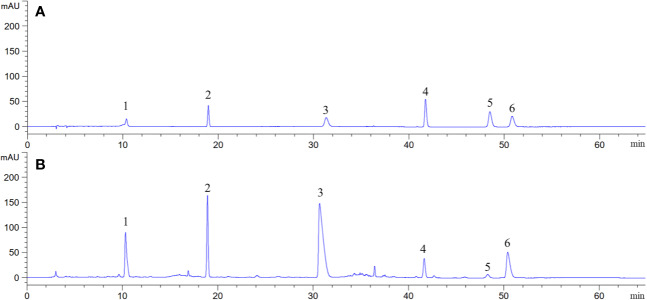
Representative HPLC chromatograms of mixed standard compounds **(A)** and *B. vernae* extract **(B)**. Peaks: 1, bufotenidine; 2, ferulic acid 4-*O*-*β*-D-glucopyranoside; 3, magnoflorine; 4, jatrorrhizine; 5, palmatine; 6, berberine.

Representative HPLC chromatogram is presented in **Figure 5**. It was found that *B. vernae* extract mainly contains six compounds (magnoflorine, bufotenidine, palmatine, ferulic acid 4-*O*-*β*-D-glucopyranoside, berberine, and jatrorrhizine). Their contents were calculated according to their calibration curves. Among the six compounds, magnoflorine showed the highest level and its content was 54.31 mg/g, followed by bufotenidine (33.40 mg/g). Moreover, the contents of ferulic acid 4-*O*-*β*-D-glucopyranoside, berberine, jatrorrhizine, and palmatine in *B. vernae* extract were determined to be 20.41, 8.93, 3.08, and 0.82 mg/g, respectively.

### Molecular Docking and CPMD Network Construction

**Figure 6:**
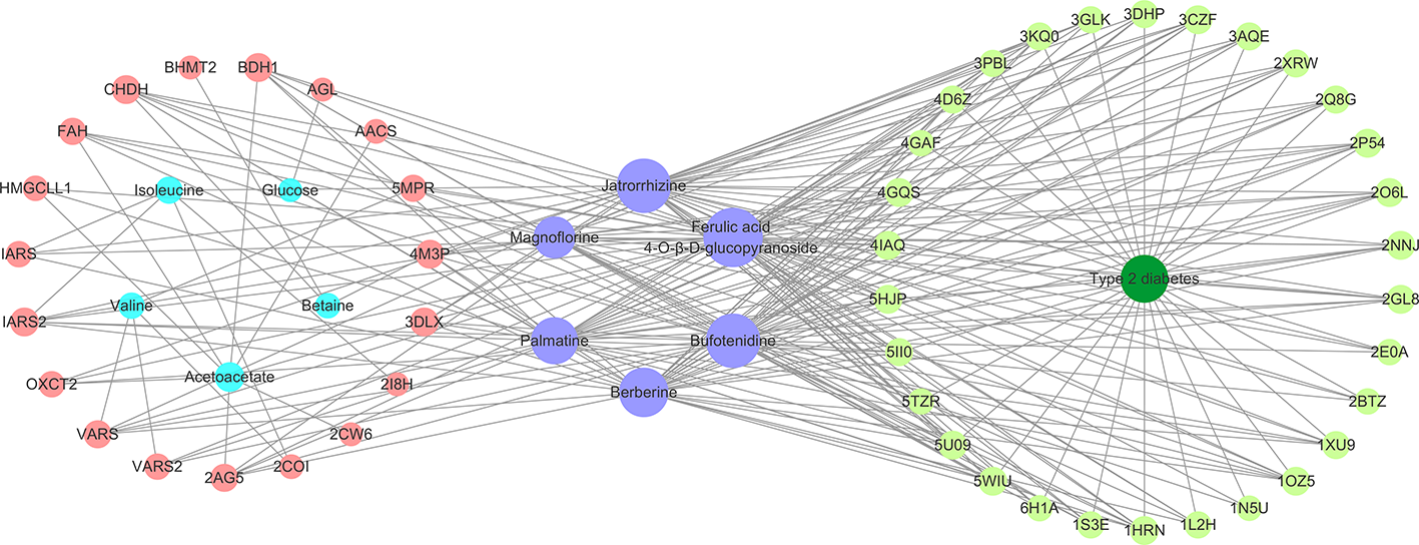
The constructed compound-protein-metabolite-disease (CPMD) interaction network revealed the correlation between chemical constituents, target proteins, differential metabolites, and type 2 diabetes. Pink and pale green circles represent target proteins related to differential metabolites and type 2 diabetes, respectively; blue, purple, and dark green represent differential metabolites, compounds, and disease, respectively.

All the six compounds meet the Lipinski’s rule. Thirty potential targets associated with type 2 diabetes were obtained from the DrugBank and TTD. Moreover, 19 candidate proteins of the above differential metabolites were obtained by Metscape. A CPMD network (**Figure 6**) based on six compounds, target proteins, differential metabolites, and type 2 diabetes was constructed to show their relevance. By analyzing the CPMD network, it was found that the same compound could target different proteins, indicating that *B. vernae* might target at biological network-level rather than target one protein. On the other hand, the same protein could be hit by different compounds, suggesting the synergistic effects of the six compounds. Moreover, the larger the purple circle representing a compound in the CPMD network, the greater the correlation between the compound and 49 target proteins. It is easy to find that ferulic acid 4-*O*-*β*-D-glucopyranoside exhibited the highest degree of correlation, followed by bufotenidine and jatrorrhizine.

Subsequently, the six compounds present in *B. vernae* extract were molecularly docked with these 49 proteins. As shown in **Table 3**, the six compounds exerted potential docking with the 44 proteins (30 disease-related proteins and 14 differential metabolites-related proteins). However, their docking capabilities were different. Ferulic acid 4-*O*-*β*-D-glucopyranoside, bufotenidine, jatrorrhizine, and berberine showed good hit rates (more than 70% of proteins with the docking scores higher than 5) for these 44 target proteins. Interestingly, similar to **Figure 6**, ferulic acid 4-*O*-*β*-D-glucopyranoside exhibited the highest hit rate (100%), followed by bufotenidine (86.36%), jatrorrhizine (86.36%), and berberine (72.73%). The results indicated that these four molecules might play a major role in the anti-diabetic effect of *B. vernae*.

**Table 3:** The number and proportion of target proteins with the absolute value of docking score more than 5.

Compounds	Docking score (the absolute value >5)	Proportion of protein (%)	PDB ID or Gene name
Ferulic acid 4-*O*-*β*-D-glucopyranoside	44	100.00	1N5U, 2NNJ, 4GQS, 2O6L, 4D6Z, 3PBL, 5WIU, 4IAQ, 1OZ5, 2P54, 5HJP, 2GL8, 3KQ0, 1S3E, 3AQE, 5II0, 3DHP, 4GAF, 1XU9, 3GLK, 3CZF, 6H1A, 5U09, 2Q8G, 2BTZ, 2E0A, 5TZR, 2XRW, 1HRN, 1L2H, 3DLX, 4M3P, 2AG5, 5MPR, AACS, BDH1, CHDH, FAH, HMGCLL1, IARS, IARS2, OXCT2, VARS, VARS2
Bufotenidine	38	86.36	1N5U, 2NNJ, 4GQS, 2O6L, 4D6Z, 5WIU, 4IAQ, 1OZ5, 2P54, 5HJP, 2GL8, 3KQ0, 1S3E, 3AQE, 5II0, 3DHP, 1XU9, 3GLK, 3CZF, 6H1A, 5U09, 2Q8G, 2BTZ, 2E0A, 5TZR, 2XRW, 1HRN, 3DLX, 4M3P, 2AG5, 5MPR, BDH1, CHDH, FAH, IARS2, OXCT2, VARS, VARS2
Jatrorrhizine	38	86.36	2NNJ, 4GQS, 2O6L, 4D6Z, 3PBL, 5WIU, 4IAQ, 1OZ5, 2P54, 5HJP, 2GL8, 3KQ0, 1S3E, 3AQE, 5II0, 3DHP, 4GAF, 1XU9, 3GLK, 3CZF, 6H1A, 5U09, 2Q8G, 2BTZ, 2E0A, 2XRW, 1HRN, 1L2H, 3DLX, 4M3P, 2AG5, 5MPR, BDH1, CHDH, IARS, IARS2, OXCT2, VARS
Berberine	32	72.73	2NNJ, 4GQS, 2O6L, 4D6Z, 3PBL, 5WIU, 4IAQ, 1OZ5, 2P54, 5HJP, 2GL8, 3KQ0, 3AQE, 5II0, 3DHP, 4GAF, 1XU9, 3CZF, 5U09, 2Q8G, 2XRW, 1HRN, 1L2H, 3DLX, 4M3P, 2AG5, BDH1, CHDH, FAH, IARS, IARS2, VARS
Palmatine	29	65.91	2NNJ, 4GQS, 2O6L, 4D6Z, 3PBL, 5WIU, 4IAQ, 1OZ5, 2P54, 5HJP, 2GL8, 3AQE, 5II0, 3DHP, 1XU9, 3CZF, 5U09, 2Q8G, 2XRW, 1HRN, 3DLX, 4M3P, 2AG5, BDH1, CHDH, HMGCLL1, IARS2, VARS, VARS2
Magnoflorine	23	52.27	2NNJ, 4GQS, 2O6L, 5WIU, 4IAQ, 1OZ5, 2P54, 5HJP, 2GL8, 3KQ0, 1S3E, 3GLK, 3CZF, 5U09, 5TZR, 1HRN, 1L2H, 3DLX, 4M3P, BDH1, CHDH, FAH, IARS2

**Figure 7:**
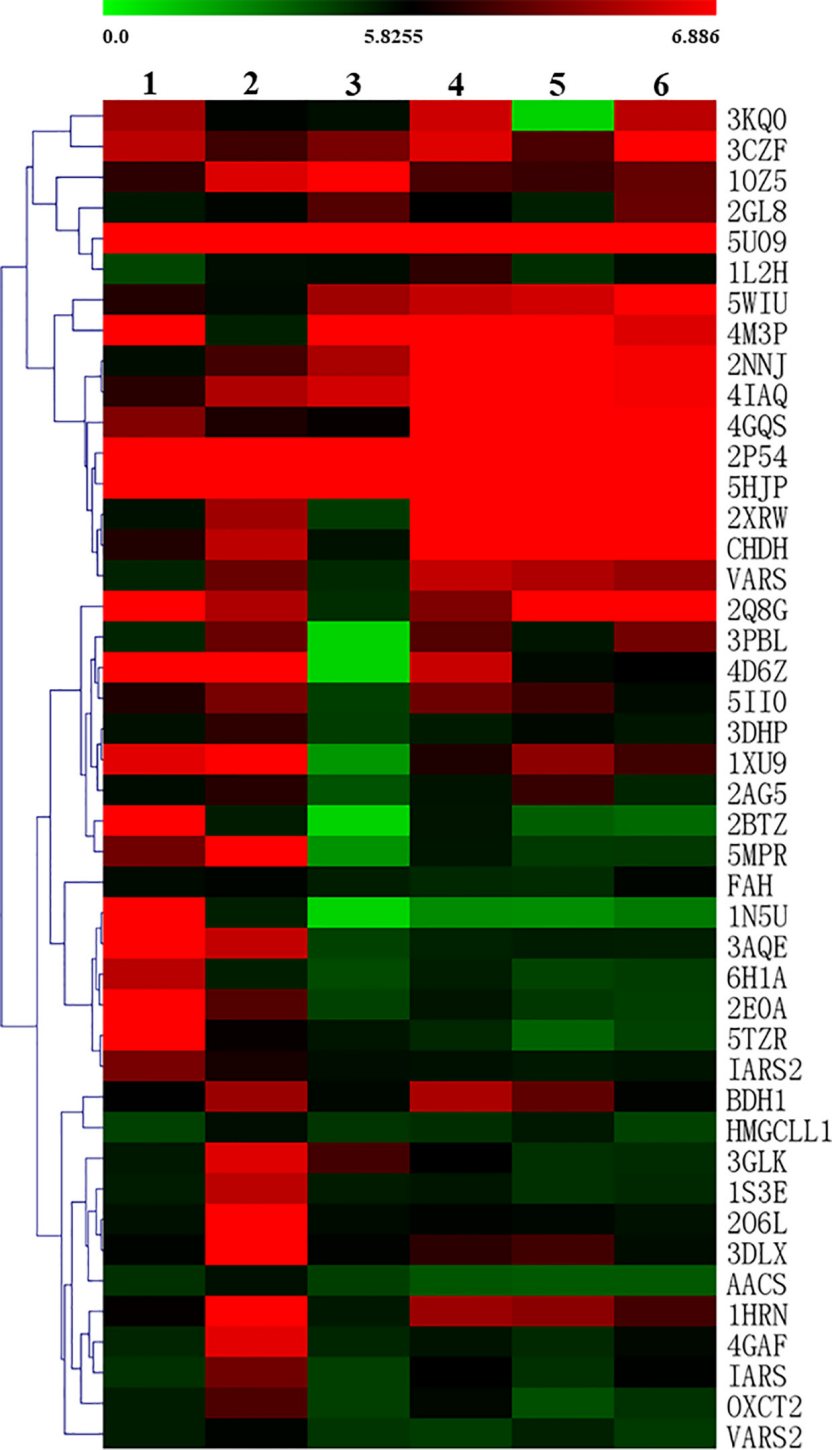
Clustering heatmap of the docking scores of the six compounds with target proteins (1, bufotenidine; 2, ferulic acid 4-*O*-*β*-D-glucopyranoside; 3, magnoflorine; 4, jatrorrhizine; 5, palmatine; 6, berberine). The intensity of color indicates docking score value, green: lowest, red: highest.

To more intuitively observe the docking of the six compounds with 44 target proteins, the clustering heatmap was generated using the docking score as input data (**Figure 7**). Forty-four target proteins were clustered into three groups. Group A showed some proteins (e.g., 5U09, 2P54, and 5HJP) that were well docked by the six compounds. Group B consisted of well-docked proteins of bufotenidine and ferulic acid 4-*O*-*β*-D-glucopyranoside, such as 3AQE and 5MPR. Group C clustered several proteins (e.g., 2O6L, 1S3E, and 4GAF) that were well docked only by ferulic acid 4-*O*-*β*-D-glucopyranoside. In addition, as shown in **Figure 7**, compared with the other five compounds, ferulic acid 4-*O*-*β*-D-glucopyranoside could be better docked with most target proteins.

### Pathway Analysis

**Figure 8:**
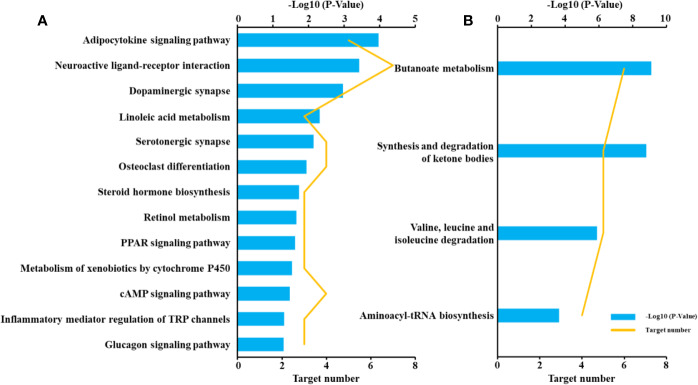
Pathway enrichment of target proteins related to type 2 diabetes **(A)** and differential metabolites **(B)**.

To reveal the molecular mechanisms of the anti-diabetic effect of *B. vernae* extract, pathway analysis of the 44 target proteins was performed. As shown in **Figure 8**, 13 pathways, such as adipocytokine signaling pathway, neuroactive ligand-receptor interaction, linoleic acid metabolism, PPAR signaling pathway, were enriched for the 30 disease-related proteins. In addition, four pathways, including synthesis and degradation of ketone bodies, butanoate metabolism, valine, leucine, and isoleucine degradation, and aminoacyl-tRNA biosynthesis, were enriched for the 14 differential metabolites-related proteins. These findings suggested that the six compounds could act on both metabolic regulation and disease-related pathways.

## Discussion

### Anti-Diabetic Effects of *B. vernae* Extract on Type 2 Diabetic Rats

In type 2 diabetes, muscle and fat cells are “resistant” to the actions of insulin and compensatory mechanisms are activated in the β-cell to secrete more insulin (Bell and Polonsky, 2001). Therefore, in this study, the INS and HOMA-IR levels in diabetic rats were significantly higher than those in normal rats. After treatment with *B. vernae* extract for 30 days, the HOMA-IR of diabetic rats was significantly decreased, while the ISI was significantly increased, indicating that *B. vernae* extract is capable of reducing insulin resistance and improving the body’s sensitivity to insulin, which may be the reason why *B. vernae* can reduce the FBG level in diabetic rats.

Previous study has shown that inflammatory participates in the pathogenesis of type 2 diabetes (Donath and Shoelson, 2011). Moreover, Hirabara et al. (2012) has demonstrated that some inflammatory factors, such as TNF-α, IL-1, and IL-6, were involved in insulin resistance. The present results revealed that *B. vernae* extract could significantly reduce the TNF-α, IL-1β, and IL-6 levels of diabetic rats, suggesting that *B. vernae* might have potential anti-inflammation activity, which is beneficial to improve the inflammatory state of type 2 diabetes.

Metabolomics is an effective method to study the holistic pharmacological effects of traditional herbal medicines *in vivo* (Peng et al., 2019). In this study, the anti-diabetic effects of *B. vernae* extract on type 2 diabetic rats were investigated for the first time using a ^1^H NMR-based metabolomics method combined with multivariate statistical analysis. As shown in **Figure 2** and **Table 2**, *B. vernae* extract could effectively restore the metabolic disorders caused by type 2 diabetes.

### Potential Active Compounds of *B. vernae* Extract Against Type 2 Diabetes

In this study, by combining molecular docking, CPMD network analysis and clustering heatmap results, we found that all the six compounds could not only regulate targets associated with endogenous metabolites, but also act on targets related to type 2 diabetes. These results are consistent with the fact that herbal medicines have multiple ingredients that can act on multiple targets. However, these six compounds were found to have different ability to interact with the target protein. Among them, bufotenidine, jatrorrhizine, berberine, and ferulic acid 4-*O*-*β*-D-glucopyranoside showed high hit rates (>70%), indicating that these four compounds might be served as the potential active ingredients of *B. vernae* against type 2 diabetes.

It is well known that alkaloids are the major bioactive compounds in *Berberis* plants (Bhardwaj and Kaushik, 2012). In the present study, three alkaloids (berberine, bufotenidine, and jatrorrhizine) were found to contribute to the anti-diabetic effect of *B. vernae* by molecular docking method. Both berberine and jatrorrhizine have been reported to show significant anti-diabetic activity by lowering glucose, improving insulin sensitivity, and stimulating insulin secretion (Fu et al., 2005; Tang et al., 2006; Pang et al., 2015). However, no anti-diabetic activity of bufotenidine has been reported, which deserves further study.

**Figure 9:**
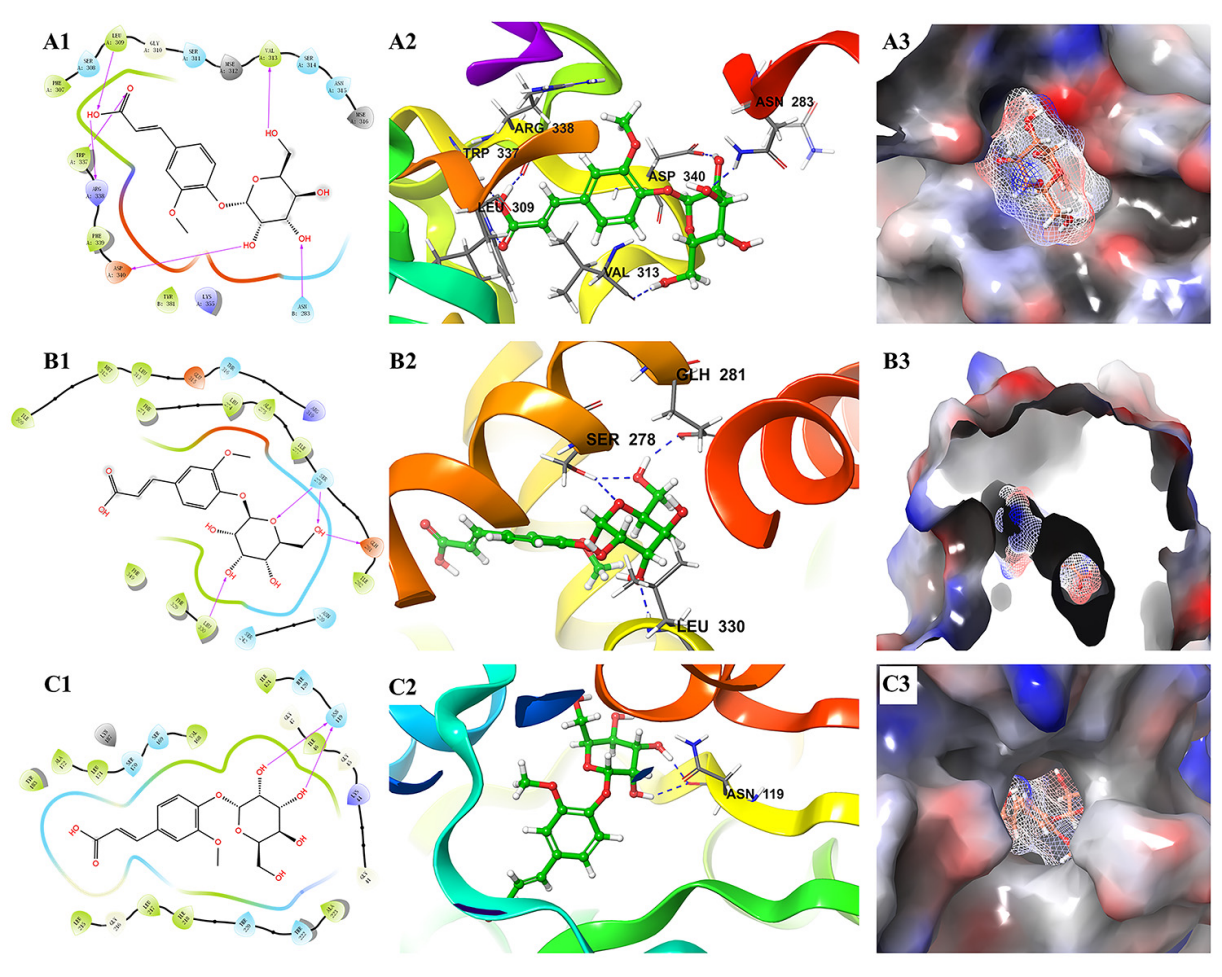
Molecular docking results of ferulic acid 4-O-*β*-D-glucopyranoside with the three proteins with the highest docking scores. **(A)** 2O6L; **(B)** 5HJP; **(C)** 1XU9. 1, the binding interaction between ferulic acid 4-*O*-*β*-D-glucopyranoside and the related target protein. The purple arrows show hydrogen bonds, capital letters and the shape of water-drop represent amino acids; 2, the binding mode of ferulic acid 4-*O*-*β*-D-glucopyranoside and amino acids in protein ribbons. Dashed lines represent hydrogen bonds; 3, charge distribution on the surface of a three-dimensional model of related target. Negative charges are depicted in red and positive charges in blue.

Moreover, it is interesting that ferulic acid 4-*O*-*β*-D-glucopyranoside could bind well with almost all 44 proteins. In particular, it docked well with some proteins (e.g., 2O6L, 1S3E, and 4GAF) that were poorly docked by the other five alkaloids. The docking results of ferulic acid 4-*O*-*β*-D-glucopyranoside with the three proteins with the highest docking scores are shown in **Figure 9**. These findings suggested that ferulic acid 4-*O*-*β*-D-glucopyranoside might be an important active compound in *B. vernae*. However, to date, there have been no reports for the anti-diabetic activity of ferulic acid 4-*O*-*β*-D-glucopyranoside. Based on the results of this study, further *in vitro* and/or *in vivo* studies are needed to determine the anti-diabetic effect of ferulic acid 4-*O*-*β*-D-glucopyranoside.

### Molecular Mechanisms of *B. vernae* Extract Against Type 2 Diabetes

In this study, molecular docking, CPMD network analysis, and pathway enrichment approaches were applied to dissect the molecular mechanisms of *B. vernae* extract against type 2 diabetes from a network-modulation point of view. The results suggested that the anti-diabetic mechanisms of *B. vernae* might be related to its regulation of several metabolic and disease-related signal pathways.

#### Regulation of Metabolic Pathways

##### Synthesis and Degradation of Ketone Bodies

Ketone bodies can replace glucose as the main energy source for brain and muscle tissues when glucose supply is insufficient (Jin, 2011). Ketone body metabolism includes two parts, namely synthesis and degradation. Firstly, lipids or VLDL/LDL can be broken down to produce free fatty acids. The fatty acids are further decomposed in the mitochondria and undergo β-oxidation to form acetyl-CoA, and then acetyl-CoA synthesizes several ketone bodies, such as acetoacetate and acetone (Jin, 2011; Tao et al., 2017). On the other hand, some ketone bodies including acetoacetate and 3-hydroxybutyrate can be converted into acetyl-CoA, which is terminally oxidized in the TCA cycle to provide energy (Cotter et al., 2013). It was reported that type 2 diabetes was accompanied by disorders of ketone body metabolism (Cotter et al., 2013). In the present study, acetoacetate (a well-known ketone body), lipids, and VLDL/LDL were observed to be decreased significantly in the serum of diabetic rats compared with those in the normal rats, indicating a disorder of ketone body metabolism in the diabetic state. After *B. vernae* extract treatment, the levels of VLDL/LDL, lipids, and acetoacetate were adjusted back to normal, indicating that *B. vernae* could regulate the synthesis and degradation of ketone bodies.

##### Butanoate Metabolism

Butanoate metabolism pathway includes many metabolites, such as acetyl-CoA, acetoacetate, pyruvate, and glutamate. Acetoacetate can be produced from fatty acids in the liver and used by the body as energy (Peng et al., 2019). In addition, glutamate can be converted into 2-oxoglutarate, which plays an important role in the TCA cycle and participates in energy metabolism (Tao et al., 2017). In this study, the lower levels of acetoacetate and glutamate were observed in the serum of diabetic rats than those in normal rats. After *B. vernae* extract intervention, the level of acetoacetate was increased, suggesting that *B. vernae* might maintain the energy balance of the body by regulating the butanoate metabolism pathway.

##### Valine, Leucine, and Isoleucine Degradation

Valine, isoleucine, and glutamate belong to glucogenic amino acids. On the one hand, their reduction may reflect the promotion of gluconeogenesis, because glycogenic amino acids can be converted to glucose through gluconeogenesis. This can be demonstrated by elevated glucose levels in the diabetic rats. On the other hand, skeletal muscle is the main storage target site for insulin-stimulated glucose uptake. In the insulin resistance state, some amino acids (e.g., valine and glutamate) need to enter the TCA cycle to produce ATP and energy for the skeletal muscle (Chao et al., 2014). Therefore, the pathway of valine, leucine, and isoleucine degradation plays an important role in glucose metabolism and energy metabolism. In the present study, *B. vernae* extract increased the levels of alanine, isoleucine, and valine after treatment, suggesting that the therapeutic effect of *B. vernae* may be related to the regulation of valine, leucine, and isoleucine degradation pathways.

#### Regulation of Disease-Related Signal Pathways

In addition to the aforementioned metabolic pathways, several disease-related signal pathways, such as adipocytokine signaling pathway, neuroactive ligand-receptor interaction, linoleic acid metabolism, PPAR signaling pathway, and inflammatory mediator regulation of TRP channels, have also been found to be regulated by *B. vernae* extract. Adipose tissue is an important endocrine organ. It can secrete various hormones (e.g., adiponectin, leptin, and resistin) and cytokines (e.g., TNF-α and IL-6). All these adipocytokines play important roles in the regulation of energy metabolism, glucose and lipid metabolism (Jaganathan et al., 2018). Li et al. (2019a) reported that four adipocytokines were closely related to the occurrence and development of diabetes. Neuroactive ligand-receptor interaction is one of the major pathways for leptin deficiency, leptin receptor deficiency, and genetic obesity (Khanal et al., 2019). German et al. (2010) found that leptin deficiency plays a key role in the pathogenesis of insulin resistance. Moreover, linoleic acid, an n-6 polyunsaturated fatty acid, is inversely associated with risk of type 2 diabetes (Zong et al., 2019). It is well known that PPARs are effective targets for treating type 2 diabetes, dyslipidemia, and obesity (Ament et al., 2012). In particular, PPAR-γ and PPAR-δ agonists have good regulatory effects on insulin resistance and glucose metabolism (Charbonnel, 2009; Ament et al., 2012). In addition, evidence suggests that TRP channels may be involved in the physiology and pathophysiology of inflammation (Parenti et al., 2016), which is an important risk factor for diabetes.

Traditional herbal medicines have multiple ingredients that can act on multiple targets. Due to the complexity of herbs, it is intractable to determine their active substances and mechanisms of action using only a single method. Therefore, an integrated strategy that can deeply understand the holistic and synergic essence of herbal medicines is necessary. In this study, ^1^H NMR metabolomics, molecular docking, and network analysis were successfully integrated to reveal the anti-diabetic effects, underlying mechanisms, and active compounds of *B. vernae*. The obtained results indicated that *B. vernae* has a significant anti-diabetic effect. The potential targets and active ingredients found in this study may provide valuable information for the quality control and drug development of *B. vernae*. Moreover, our study provides a new methodological reference for revealing the active ingredients and regulatory mechanisms of complex herbal medicines.

## Conclusion

*B. vernae* showcased great potential for treating type 2 diabetes. It could improve insulin resistance and inflammation, reduce serum glucose, and increase insulin sensitivity in type 2 diabetic rats. Moreover, diabetes-induced disturbances to the metabolic profile were partially reversed by *B. vernae* treatment. An integrated pathway analysis revealed that several metabolic pathways (e.g., butanoate metabolism) and disease-related signal pathways (e.g., adipocytokine signaling pathway) were significantly associated with the anti-diabetic effects of *B. vernae*. In addition, four compounds (ferulic acid 4-*O*-*β*-D-glucopyranoside, bufotenidine, jatrorrhizine, and berberine) were found to dock well with the targets both associated with endogenous metabolites and type 2 diabetes. They are considered to be potential active ingredients of *B. vernae* against type 2 diabetes. Further investigations are needed to verify the anti-diabetic effects of ferulic acid 4-*O*-*β*-D-glucopyranoside and bufotenidine.

## Data Availability Statement

The raw data supporting the conclusions of this article will be made available by the authors, without undue reservation, to any qualified researcher.

## Ethics Statement

The animal study was reviewed and approved by the Animal Ethics Committee of Chengdu University of Traditional Chinese Medicine.

## Author Contributions

QL conducted the experiments, performed data analysis, and wrote the paper. CZ, YuZ, HD, TX, and XX participated in the experimental process. JZ, TK, XL, and YiZ supported the study and critically revised the paper. GF conceived and designed the study. All authors contributed to the article and approved the submitted version.

## Conflict of Interest

The authors declare that the research was conducted in the absence of any commercial or financial relationships that could be construed as a potential conﬂict of interest.
